# Dose perturbation due to the polysulfone cap surrounding a Fletcher‐Williamson colpostat

**DOI:** 10.1120/jacmp.v11i1.3146

**Published:** 2010-01-28

**Authors:** Michael J. Price, Stephen F. Kry, Patricia J. Eifel, Mohammad Salehpour, Firas Mourtada

**Affiliations:** ^1^ Department of Medical Physics Mary Bird Perkins Cancer Center and Department of Physics and Astronomy Louisiana State University Baton Rouge LA USA; ^2^ Department of Radiation Physics The University of Texas M.D. Anderson Cancer Center Houston TX USA; ^3^ Department of Radiation Oncology The University of Texas M.D. Anderson Cancer Center Houston TX USA

**Keywords:** cervical cancer, brachytherapy, Fletcher‐Williamson colpostat, Ovoid, polysulfone cap, vagina mucosa dose

## Abstract

We conducted a Monte Carlo evaluation of the dosimetric impact of the polysulfone cap used with the Fletcher‐Williamson (FW) colpostat for I192r high‐dose rate and pulsed‐dose rate intracavitary brachytherapy. Polysulfone caps with diameters of 30 mm, 25 mm, 20 mm, and 16 mm (mini‐ovoid) were simulated, and the absorbed dose rate in surrounding water was calculated and compared to the dose rate calculated for a bare I192r source in water. The dose perturbation depended on the cap diameter, distance away from the cap surface, and angular position around the cap. The largest dose rate reductions were found to be where the cap is thickest, in the general direction of the tumor bed. The range of perturbation mainly from the polysulfone cap over all depths and cap diameters was +2.8% (dose enhancement) to −6.8% (dose reduction); this perturbation is separate from the well‐documented attenuation of the rectal and bladder tungsten shields of the colpostat. Based on the impact of the polysulfone cape, the FW colpostat cap's material composition should be modified to reduce this dosimetric effect, or brachytherapy treatment planning dose algorithms should be improved to account for this perturbation.

PACS numbers: 87.10.Rt 87.53.‐j 87.53.Bn 87.53.Jw

## I. INTRODUCTION

Intracavitary brachytherapy is a standard treatment for loco‐regionally advanced cervical cancers, with a majority of patients receiving both external beam radiotherapy and intracavitary brachytherapy.^(^ ^1^ ^–^ ^3^ ^)^ Modern intracavitary brachytherapy practice typically uses high‐dose rate (HDR) or pulsed‐dose rate (PDR) techniques with afterloading technology. For example, both Nucletron HDR and PDR brachytherapy systems (Nucletron Corporation, Veenendaal, The Netherlands) use a pair of Fletcher‐Williamson (FW) colpostats and an intrauterine tandem to facilitate the delivery of radiation from an I192r source via an afterloader. The colpostats serve to deliver a prescribed dose of radiation to diseased tissue while concurrently displacing healthy tissue away from the radioactive source. Colpostats can be adjusted in size to accommodate a range of patient anatomy, which is achieved by using a variety of cap sizes of between 16 and 30 mm in diameter. These caps are composed of polysulfone, but do not completely fill the colpostat and thereby also include air cavities.

Commercial brachytherapy treatment planning systems utilizing American Association of Physicists in Medicine Task Group 43 parameters^(^ ^4^ ^,^ ^5^ ^)^ assume that the radioactive source is surrounded by homogeneous water. While this may be a reasonable assumption for tissue, it may lead to erroneous dose calculations when colpostats with polysulfone caps are used, as polysulfone has a higher density than water and air has a much lower density.

Ye et al.^(6)^ found that applicators composed of materials of nonunity density could impact the dose predicted by the treatment planning system. They reported the attenuating effects of a stainless steel uterine tube applicator and vaginal cylinder applicators consisting of uterine tubes surrounded by polysulfone sleeves of 20 mm and 40 mm in diameter. They found that a 5% improvement in accuracy of dose delivered to the patient using an I192rHDR vaginal cylinder could be achieved by compensating for the attenuation of the applicator system during treatment planning system calculations.^(6)^ Ye et al. also found that the composition of an applicator may perturb the dose distribution. However, their work did not examine perturbations associated with FW colpostats, which could be substantial, given the size of some colpostats. This, along with the ubiquitous use of such colpostats, requires that a dosimetric examination be conducted.

The current study is an evaluation of the impact on the dose of polysulfone caps in Fletcher‐Williamson colpostats as compared to the dose predicted by a water‐equivalent environment.

## II. MATERIALS AND METHODS

### A. I192r source and Fletcher‐Williamson colpostats model descriptions

**Figure 1:**
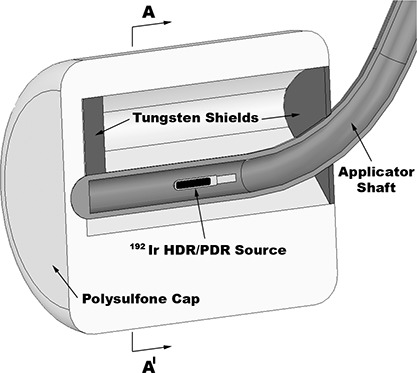
Components of the Nucletron Fletcher‐Williamson high‐dose rate (HDR) and pulsed‐dose rate (PDR) colpostat. Shown is a plane cut along the colpostat long axis with the intercolpostat shields (rectal shield [left], bladder shield [right]), the polysulfone cap, and the iridium‐192 (I192r) HDR/PDR source.

Figure 1 depicts a central cut of the FW colpostat and the mHDR v2 I192r source centered in the colpostat. The active length and diameter of the I192r source are 3.6 mm and 0.65 mm (mHDR v2), respectively. The iridium core of the source is encapsulated in an AISI 316L stainless steel cylinder with a rounded tip. Further details of the mHDR v2 source model can be found in the literature.^(^ ^7^ ^–^ ^9^ ^)^

**Figure 2:**
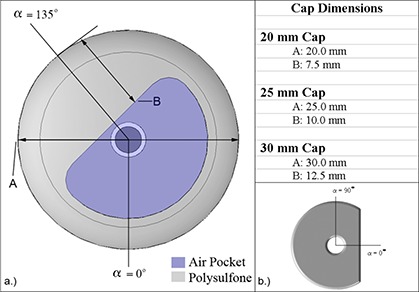
Components of the Nucletron Fletcher‐Williamson high‐dose rate (HDR) and pulsed‐dose rate (PDR) colpostat. Shown is a plane cut along the transverse cross‐section through plane ${\text{AA}}^{I}$. The simulation angles of α=0° and α=135° for the standard Fletcher‐Williamson caps (a) and α=0° and α 90° for the minicolpostat cap (b) are defined.

The major components of the FW colpostat, as detailed by Price et al.,^(9)^ are an AISI 316L stainless steel tube, a retaining screw, air within the colpostat and applicator tube, and rectal and bladder shields (modeled as Densimet 17). Separate polysulfone cap models were created for each of the three cap sizes used clinically at our institution (20 mm, 25 mm, and 30 mm diameters) (Fig. 2). The density of polysulfone is stated by the vendor as $\rho =1.24\;g\;{\text{cm}}^{-3}$. However, Ye et al. reported a range of values for polysulfone density from $1.24\;g\;{\text{cm}}^{-3}$ to $1.6\;g\;{\text{cm}}^{-3}$, and measured a density of $\rho =1.40\;g\;{\text{cm}}^{-3}$. In our laboratory, we measured the density of polysulfone to be $\rho =1.38\;g\;{\text{cm}}^{-3}$ and this value was used for all Monte Carlo simulations. The polysulfone composition was modeled as 50.5% carbon, 34.5% hydrogen, 10.0% oxygen, and 5.0% sulphur (by weight).^(9)^

**Figure 3:**
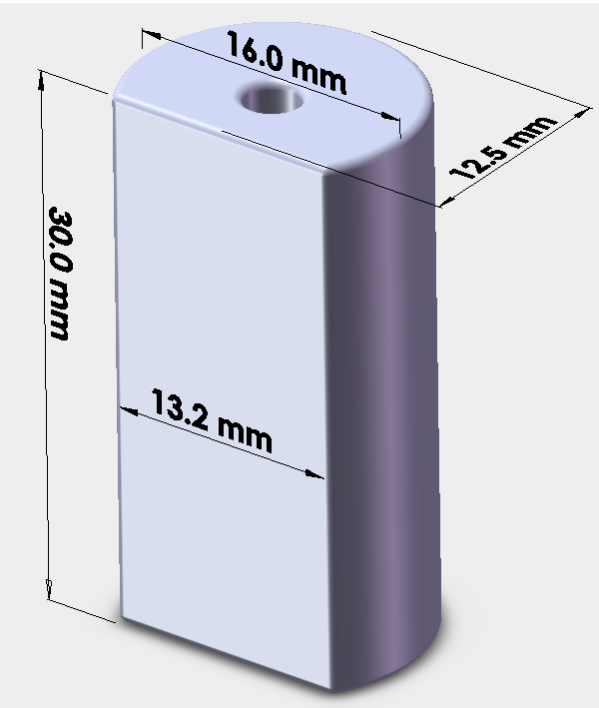
Dimensions of the Nucletron minicolpostat polysulfone cap (16 mm diameter) used for the Monte Carlo simulations.

In addition to the FW models described above, we constructed a MC model of Nucletron's minicolpostat intracavitary brachytherapy applicator. This applicator is 16 mm in diameter and does not contain intercolpostat shields. The dimensions of the minicolpostat polysulfone cap are shown in Fig. 3.

For all MC simulations, the HDR/PDR I192r source was centered within, and with respect to, the long axis of the stainless steel applicator tube. To preserve backscatter, the colpostat was placed at the center of a water sphere with a diameter of 30 cm.^(^ ^7^ ^,^ ^10^ ^)^

### B. Monte Carlo simulations

The current study used a previously validated Monte Carlo model (MCNPX version 2.6a^(11)^ of the Nucletron HDR/PDR I192r source and Fletcher‐Williamson (FW) colpostat.^(9)^ Photon transport was simulated in the models using the Evaluated Nuclear Data File (ENDF/B‐VI) library. The dose to water around a single colpostat was estimated by tallying the photon kerma rate. This rate, calculated based on a track‐length estimate of the photon flux, has been shown to be a reasonable approximation of dose.^(^ ^7^ ^–^ ^9^ ^)^ Photons were tracked to an energy of 1 keV. The I192r spectrum, reported by Glasgow and Dillman^(12)^ and used in the literature,^(^ ^7^ ^,^ ^9^ ^)^ was used to perform all Monte Carlo simulations.

MCNPX kerma tallies were done in one cylindrical plane whose origin coincided with the midpoint of the long axis of the HDR/PDR I192r source. The cylindrical plane had a thickness of 2 mm, extended outward radially 9 cm, and was divided into voxels defined by Δα=5° and XXDr=1mm. The 0° angle was defined as shown in Fig. 2, and all reported distances were relative to the outer edge of the polysulfone cap. All MC simulations were run with $2\times 10^{9}$ histories. The dose rate per photon calculated by the MC simulations was converted to dose rate per hour $\left(\text{cGy}\;h^{-1}\right)$, following the methodology described by Price et al.^(13)^

The dose rate per hour was calculated for the HDR/PDR I192r source in a single colpostat with different polysulfone cap diameters (30 mm, 25 mm, 20 mm, and 16 mm [minicolpostat]). The dose rate per hour was also calculated by the Monte Carlo model for a bare mHDR v2 source in pure water for comparison. This comparison reflects the true dose rate as compared to that predicted by the treatment planning system using TG‐43 formalism along the bisecting axis of the colpostat. The expanded uncertainty of these calculations was ±1.2% for k=2, approximating the 95% confidence interval.^(14)^

## III. RESULTS & DISCUSSION

**Figure 4:**
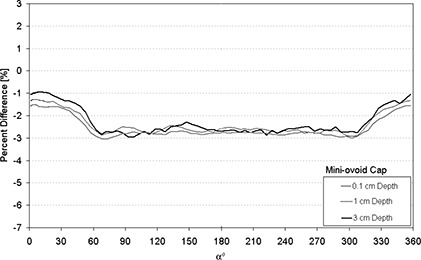
Percent difference between dose rate predicted by MC modeling for an HDR/PDR I192r source with a minicolpostat polysulfone cap relative to the same source in water.

**Figure 5:**
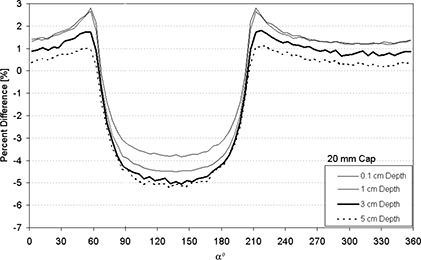
Percent difference between dose rate predicted by Monte Carlo (MC) modeling for an HDR/PDR I192r source with a 20 mm polysulfone cap relative to the same source in water.

**Figure 6:**
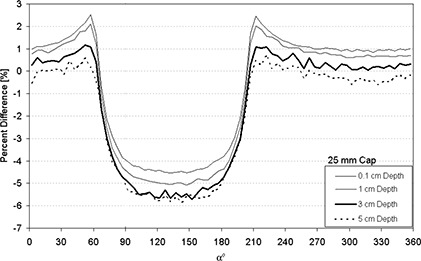
Percent difference between dose rate predicted by MC modeling for an HDR/PDR I192r source with a 25 mm polysulfone cap relative to the same source in water.

**Figure 7:**
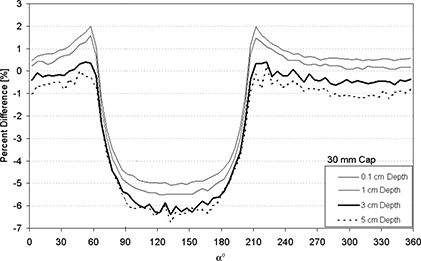
Percent difference between dose rate predicted by MC modeling for an HDR/PDR I192r source with a 30 mm polysulfone cap relative to the same source in water.

Figures 4–7 display the percent absolute dose rate difference for a HDR/PDR I192r source centered within a minicolpostat, 20 mm, 25 mm, or 30 mm polysulfone cap relative to dose rate from a bare source in water. The plots are shown for 0.1 cm, 1 cm, 3 cm, and 5 cm radial distance away from the surface of the polysulfone cap.

For the minicolpostat (Fig. 4), the dose perturbation was relatively uniform, being approximately 2.5% less than the dose from the bare source in water regardless of angle or distance from the colpostat surface. The dose is reduced because of the higher density of polysulfone relative to water, and is uniform with angle because of the structural uniformity of the cap (Fig. 3).

For the 20 mm, 25 mm, 30 mm diameter caps, a more substantial and less uniform perturbation was observed (Figs. 5–7). Most dramatically, and for nearly 180°, the presence of the polysulfone cap reduced the dose relative to the dose from the bare source in water (negative percent difference). This dose perturbation was larger for larger caps, and was greatest at approximately 135° (see Fig. 2). Both of these observations can be understood in terms of the thickness of polysulfone through which the photons must traverse, which is increased for larger caps and at approximately equal to 135°. In a clinical situation, this direction corresponds approximately to the direction of the tumor bed, which is undesirable to underdose. In contrast, there were spans of α for which the inclusion of the polysulfone cap resulted in a higher dose than the standard TPS prediction using the source in water. This case developed because, in approximately half of the full‐size polysulfone cap, air is the predominant component (Fig. 2). As air is less attenuative to radiation than water, there is actually more dose delivered than predicted by a conventional treatment planning system by 2–3%. Clinically, the location of this overdosing is most likely to be in the packing material that is placed within the vagina by the clinician to retain the position of the inserted applicator and displace the rectum and bladder away from the activity contained within the colpostats.

**Figure 8:**
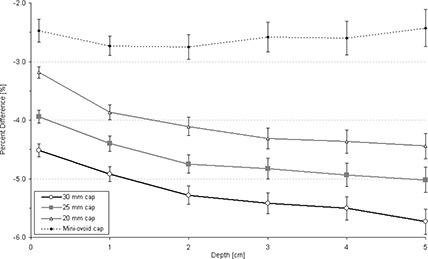
Percent difference between dose rate predicted by MC modeling for an HDR/PDR I192r source with a minicolpostat polysulfone cap relative to the same source in water as a function of depth.

Figure 8 displays the dose rate perturbation as a function of distance from the cap surface at an angle of 135°. For all cap sizes except the mini‐ovoids, there is a falloff in dose delivered with respect to depth.

Table 1 summarizes the largest and average calculated positive and negative percent dose differences. A positive percent dose difference means there is an increased dose when the I192r source is placed inside a colpostat relative to a bare I192r source in water, while a negative percent dose indicates a decreased dose. For all investigated cases, the largest reduction in dose was −6.8% at 4 cm depth when the 30 mm polysulfone cap was used.

**Table 1:** Percent difference for all polysulfone cap sizes and depths. The columns tabulating the average positive and negative percent differences are calculated by averaging all of the respective positive and negative percent differences with respect to polysulfone cap size.

*Cap Size (mm)*	*Depth (cm)*	*Largest Positive %*Δ *(%)*	*Largest Negative %*Δ *(%)*	*Average Positive %*Δ *(%)*	*Average Negative %*Δ *(%)*
20	0.1	2.8	−3.8	1.6	−3.7
20	1	2.7	−4.5	1.6	−3.7
20	2	2.2	−4.8	1.3	−3.9
20	3	1.8	−5.0	1.1	−4.1
20	4	1.4	−5.1	0.8	−4.2
20	5	1.1	−5.2	0.5	−4.3
20	6	0.9	−5.3	0.4	−3.9
25	0.1	2.5	−4.5	1.3	−3.7
25	1	2.1	−5.1	1.0	−4.2
25	2	1.6	−5.4	0.7	−4.5
25	3	1.2	−5.7	0.5	−4.7
25	4	0.8	−5.9	0.3	−3.2
25	5	0.7	−5.8	0.2	−2.7
25	6	0.4	−6.0	0.2	−2.3
30	0.1	2.0	−5.1	0.8	−4.2
30	1	1.6	−5.5	0.5	−4.7
30	2	1.0	−6.0	0.4	−3.0
30	3	0.4	−6.4	0.3	−2.6
30	4	0.2	−6.8	0.1	−2.5
30	5	0.2	−6.7	0.2	−2.6
30	6	0.0	−6.7	0.0	−2.7
mini	0.1	1.0	−3.1	0.0	−2.4
mini	1	1.3	−3.3	0.0	−2.6
mini	2	1.0	−3.2	0.0	−2.4
mini	3	0.7	−3.3	0.0	−2.3
mini	4	0.0	−3.6	0.0	−2.3
mini	5	0.8	−3.8	0.0	−2.4
mini	6	0.8	−3.7	0.0	−2.3

The findings of this study provide an appreciation of the magnitude of the impact that polysulfone caps have on dose delivered outside the cap relative to the approximations made by treatment planning systems. These dose perturbations were based on the impact of the polysulfone cap, stainless steel tube, and associated air pockets, and were not an evaluation of the impact of tungsten shields. Although these shields were included in the Monte Carlo model, the dose was evaluated only in a plane bisecting the center of the colpostat, and therefore the tungsten shields did not directly affect the dose distributions presented in the results of this study.

This study did not evaluate the impact of the polysulfone caps on the dose in different planes, or consider the impact of multiple source dwell positions. Consequently, this work does not capture the variety of perturbations that would be associated with a clinical situation. However, this study identifies that these caps do indeed have an impact on the dose, and attention should be given to the dosimetric perturbations they could induce in clinical situations. This is particularly true as the dose perturbations are likely to be slightly larger in clinical situations. The current study examined the dose from gamma rays normally incident on the polysulfone cap (a source in the center of the colpostat). In clinical situations, the use of multiple dwell positions with result in gamma rays with oblique angles of incidence on the cap. Such oblique angles of incidence would correspond to longer path lengths through the polysulfone (or air) as compared to normal incidence, thereby increasing the effect of the perturbation. This effect was observed by Ye et al.^(6)^ who found an increased perturbation of 0.6% (a 15% relative increase) when considering nine I192rHDR source dwell positions within a heterogeneous vaginal cylinder applicator when compared to a single source dwell position.

The American Association of Physicists in Medicine Task Group 56 discusses in detail the sources of errors compromising dose delivery accuracy, which is divided into physical and clinical aspects.^(15)^ Physically accurate dose delivery is achieved if the predicted dose and actual dose absorbed by the medium are equal at reference points in tissue. Several factors including the accuracy of source strength, physics data of the source, and the influence of applicator attenuation are identified as main contributors to the physical uncertainty. It is expected that dose delivery accuracy on the order of 5–10% is achievable at distances of 1–5 cm for most brachytherapy sources. Accounting for the polysulfone dose perturbation is therefore expected to improve the HDR/PDR dose delivery accuracy.

## IV. CONCLUSIONS

It was found that the structure and composition of the polysulfone cap in the FW colpostat could substantially perturb the dose delivered to the surrounding medium as compared to assuming the colpostat is water equivalent. This dosimetric impact could exceed 5% for the Monte Carlo study conducted here, and could readily be larger in clinical situations. These results suggest that the colpostat material and composition should be accounted for in patient dose calculations. This could be done by modifying the current FW HDR/PDR colpostat cap material to reduce this dosimetric error, or by improving brachytherapy TPS dose algorithms so they can account for the perturbation caused by this and other applicator structures.

## ACKNOWLEDGEMENTS

This research was partially supported by the Nucletron Corporation.

## References

[1] Fletcher GH , Hamberger AD . Squamous cell carcinoma of the uterine cervix. In: Fletcher GH (ed.) Textbook of Radiotherapy. 3rd ed. Philadelphia, PA: Lea & Febiger; 1980.

[2] Katz A , Eifel PJ . Quantification of intracavitary brachytherapy parameters and correlation with outcome in patients with carcinoma of the cervix. Int J Radiat Oncol Biol Phys. 2000;48(5):1417–25. 11121642 10.1016/s0360-3016(00)01364-x

[3] Nag S , Erickson B , Thomadsen B , Orton C , Demanes JD , Petereit D . The American Brachytherapy Society recommendations for high‐dose‐rate brachytherapy for carcinoma of the cervix. Int J Radiat Oncol Biol Phys. 2000;48(1):201–11. 10924990 10.1016/s0360-3016(00)00497-1

[4] Nath R , Anderson LL , Luxton G , Weaver KA , Williamson JF , Meigooni AS . Dosimetry of interstitial brachytherapy sources: recommendations of the AAPM Radiation Therapy Committee Task Group No. 43. American Association of Physicists in Medicine. Med Phys. 1995;22(2):209–34. 7565352 10.1118/1.597458

[5] Rivard MJ , Coursey BM , DeWerd LA , et al. Update of AAPM Task Group No. 43 Report: a revised AAPM protocol for brachytherapy dose calculations. Med Phys. 2004;31(3):633–74. 15070264 10.1118/1.1646040

[6] Ye SJ , Brezovich IA , Shen S , Duan J , Popple RA , Pareek PN . Attenuation of intracavitary applicators in 192Ir‐HDR brachytherapy. Med Phys. 2004;31(7):2097–106. 15305463 10.1118/1.1762791

[7] Daskalov GM , Loffler E , Williamson JF . Monte Carlo‐aided dosimetry of a new high dose‐rate brachytherapy source. Med Phys. 1998;25(11):2200–08. 9829246 10.1118/1.598418

[8] Williamson JF , Li Z . Monte Carlo aided dosimetry of the microselectron pulsed and high dose‐rate 192Ir sources. Med Phys. 1995;22(6):809–19. 7565372 10.1118/1.597483

[9] Price MJ , Horton JL , Gifford KA , et al. Dosimetric evaluation of the Fletcher‐Williamson ovoid for pulsed‐dose‐rate brachytherapy: a Monte Carlo study. Phys Med Biol. 2005;50(21):5075–87. 16237242 10.1088/0031-9155/50/21/009

[10] Karaiskos P , Angelopoulos A , Pantelis E , et al. Monte Carlo dosimetry of a new 192Ir pulsed dose rate brachytherapy source. Med Phys. 2003;30(1):9–16. 12557972 10.1118/1.1524168

[11] Waters LS . MCNPX ‐ User's Manual Version 2.4.0. Los Alamos, NM: Los Alamos National Laboratory; 2002.

[12] Glasgow GP , Dillman LT . Specific gamma‐ray constant and exposure rate constant of 192Ir. Med Phys. 1979;6(1):49–52. 440232 10.1118/1.594551

[13] Price MJ , Gifford KA , Horton J , Lawyer A , Eifel P , Mourtada F . Comparison of dose distributions around the pulsed‐dose‐rate Fletcher‐Williamson and the low‐dose‐rate Fletcher‐Suit‐Delclos ovoids: a Monte Carlo study. Phys Med Biol. 2006;51(16):4083–94. 16885626 10.1088/0031-9155/51/16/014

[14] Taylor BN , Kuyatt CE . Guidelines for evaluating and expressing the uncertainty of NIST measurement results, Washington, DC: U.S. Government Printing Office; 1994.

[15] Nath R , Anderson LL , Meli JA , Olch AJ , Stitt JA , Williamson JF . Code of practice for brachytherapy physics: report of the AAPM Radiation Therapy Committee Task Group No. 56. American Association of Physicists in Medicine. Med Phys. 1997;24(10):1557–98. 9350711 10.1118/1.597966

